# Neural Correlates of Hate

**DOI:** 10.1371/journal.pone.0003556

**Published:** 2008-10-29

**Authors:** Semir Zeki, John Paul Romaya

**Affiliations:** Wellcome Laboratory of Neurobiology, Department of Cell and Developmental Biology, University College London, London, United Kingdom; Victoria University of Wellington, New Zealand

## Abstract

In this work, we address an important but unexplored topic, namely the neural correlates of hate. In a block-design fMRI study, we scanned 17 normal human subjects while they viewed the face of a person they hated and also faces of acquaintances for whom they had neutral feelings. A hate score was obtained for the object of hate for each subject and this was used as a covariate in a between-subject random effects analysis. Viewing a hated face resulted in increased activity in the medial frontal gyrus, right putamen, bilaterally in premotor cortex, in the frontal pole and bilaterally in the medial insula. We also found three areas where activation correlated linearly with the declared level of hatred, the right insula, right premotor cortex and the right fronto-medial gyrus. One area of deactivation was found in the right superior frontal gyrus. The study thus shows that there is a unique pattern of activity in the brain in the context of hate. Though distinct from the pattern of activity that correlates with romantic love, this pattern nevertheless shares two areas with the latter, namely the putamen and the insula.

## Introduction

In pursuing our studies of the affective states generated by visual inputs, we concentrate in this work on the sentiment of hate. Like romantic and maternal love which we reported on previously (Bartels and Zeki 2001, 2004) [1], [2], hate is a complex biological sentiment which throughout history has impelled individuals to heroic as well as evil deeds. Unlike romantic love, it need not be directed against an individual; it may instead assume many varieties, being directed against an individual, a society, or an ethnic group. In this study, we were interested to explore the neural correlates of hate directed against an individual. There are varieties even within such a confine. The hatred may be directed against a public figure or a personally known individual, for a variety of reasons. We made no attempt to distinguish between different types of personal hatred. Instead, we recruited subjects through advertisements, asking them only to volunteer if they experienced an intense enough hate for an individual, without distinguishing further between different categories of individual hate. We conformed as much as possible to our previous studies on romantic and maternal love, asking subjects to complete a questionnaire which allowed us to correlate the declared subjective experiences with changes in the blood oxygen level dependent (BOLD) signal. We hypothesized that the pattern of activity generated by viewing the face of a hated person would be quite distinct from that produced by viewing the face of a lover. In particular, we did not anticipate activation of the brain's reward system but believed that it would result in a different pattern of activity within the emotional brain. Given the common association between love and hate, and the relative frequency with which one of these sentiments can transform into the other, we also hypothesized that there would be some strong correlation in the brain sites activated during the experience of these two antipodean sentiments. The results surprised us.

## Materials and Methods

17 healthy subjects (10 male, 12 right handed, mean age 34.8 years) were recruited through advertisements. Informed written consent was obtained from all participants and the study was approved by the joint Research Ethics Committee for the National Hospital for Neurology and Neurosurgery and the Institute of Neurology. Only subjects expressing a strong hatred for an individual were selected. With one exception, all our subjects testified to the hatred of an individual, either an ex-lover or a competitor at work. The one exception was a female who expressed an intense hatred of a very famous political figure. During a primary visit to the laboratory, some two weeks prior to scanning, each subject provided picture portraits of the hated person and of three other people of the same sex towards whom they had neutral feelings, all pictures being matched as far as possible for expression and general appearance. The nature of the experiment was explained to the subject and an example stimulus using random anonymous faces was demonstrated. Subjects also completed a questionnaire during the first session to assess their feelings about the hated person and obtain a hate score which could subsequently be used as a covariate in the second level analysis (see below).

Once during the first visit and once directly after the scanning session, we tried to assess each subject's feelings about the hated person. We did so by asking them to complete a score sheet that we devised, the Passionate Hate Scale (PHS). This has a certain similarity to the Passionate Love Scale (Hatfield and Sprecher 1986 [3]) that we used in our study of romantic love. We based the PHS partially on Sternberg's (2004) [4] triangular theory of hate and on the assumption, derived from numerous studies, that there is a good correlation between declared subjective mental states, including emotional ones, and the observed BOLD signal (e.g. Kawabata and Zeki 2004) [5]. The questionnaire revolved around three elements of hate: (a) negation of intimacy, when an individual seeks a distance from the hated person. This is usually because the hated person arouses feeling of revulsion and disgust, exactly the opposite of the desire for greater intimacy in the context of love; (b) passion, expressing itself in intense anger at, and fear of, the hated person; and (c) devaluation of the hated person through expressions of contempt. Three negative statements and one positive statement were constructed around each of these elements. Subjects were invited to indicate their level of agreement with each statement, the questions being presented in a different random order on both occasions (for full details see Supporting Information File S1: Hate Questionnaire). The questionnaire could yield a “hate score” ranging from 0 (minimum hate) to 72 (maximum hate).

### Stimuli

**Figure 1:**
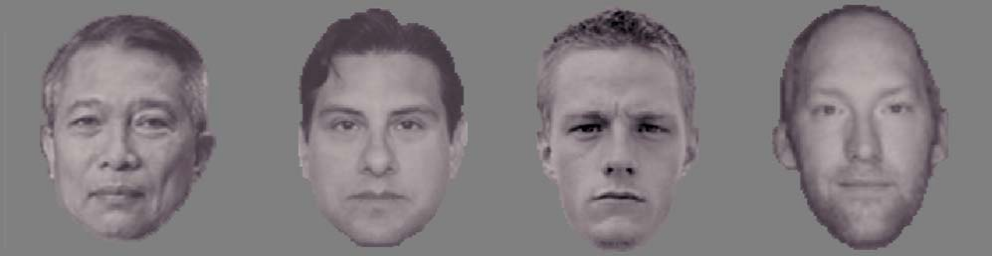
An example set of four processed face images (faces not from this study). The images are converted to greyscale and normalised with respect to visual area and average brightness. They are roughly matched in terms of spatial frequency and intensity contrast. The faces are all of the same sex, the expressions are similar and a vertically aligned full face image has been selected in each case. An individual set of four such faces was presented to each subject. One of the faces was of a person hated by that particular subject, the other three faces were known to the subject, but were of a neutral relationship, neither loved nor hated.

Stimuli were generated using Cogent 2000 and Cogent Graphics (http://www.vislab.ucl.ac.uk/Cogent2000, http://www.vislab.ucl.ac.uk/CogentGraphics). Four images provided by each subject were digitised and an image-editing program (Adobe® Photoshop® CS2) was used to remove any superfluous features such as earrings, scarves etc. The background detail was replaced with a flat mid-grey tone and the images were normalised in terms of spatial frequency, visual area, average brightness and contrast (see Figure 1). Full details on the preparation of the stimuli are given in Supporting Information file S1: Processing of face images.

Each subject was exposed to either two or three identical stimulus sessions. The session began with a flat grey background (intensity 9.06 cd/m^2^) (blank condition) which was present for 20 s during which the first six brain volumes were discarded to allow T1 equilibration effects to subside. A face was then presented for 16.07 s followed by another blank interval of 2.07 s. Occasionally, a blank condition of 16.07 s was displayed instead of a face, to increase the proportion of baseline acquisition during the scanning session. Subjects were instructed to press a key each time a face disappeared. The faces and blanks were presented in a pseudo-random, symmetrically balanced sequence (see Supporting Information file S1:Stimulus Design). The session ended with a terminal blank period of 30 s, during which the scanner continued to acquire decaying BOLD signal. A block design incorporating null events with ca. 16 s epochs was chosen for direct comparison with our previous studies on romantic and maternal love [1], [2].

### Scanning details

Scanning was done in a 1.5-T Siemens Magneton Sonata MRI scanner fitted with a head volume coil (Siemens, Erlangen, Germany) to which an angled mirror was attached, allowing subjects to view a screen onto which stimuli were projected using an LCD projector. An echo-planar imaging (EPI) sequence was applied for functional scans, measuring BOLD signals (echo time TE = 50 mS, repeat time TR = 90 ms, volume time 3.42 s). Each brain image was acquired in a descending sequence comprising 38 axial slices each 2 mm thick with an interstitial gap of 1 mm and a voxel resolution of 3×3×3 mm, covering nearly the whole brain. After functional scanning had been completed a T1 mdeft anatomical scan was acquired in the saggital plane to obtain a high resolution structural image (176 slices per volume, constant isotropic resolution 1×1×1 mm, TE = 3.56 s, TR = 12.24 s).

### Analysis

Data were analysed using SPM5 [6] (Statistical Parametric Mapping V5 http://www.fil.ion.ucl.ac.uk/spm). The time series of functional brain volume images for each subject was realigned and normalized into MNI (Montreal Neurological Institute) space [7] and then smoothed using a Gaussian smoothing kernel of 9×9×9 mm.

The stimulus for each subject was modelled as a set of regressors in the SPM5 general linear model (GLM) (first-level) analysis. The stimulus was a block design and boxcar functions were used to define regressors which modelled the onsets and durations of the appearances of each of the neutral faces and the hated face. Keypresses were modelled as delta functions in an additional regressor. Head movement parameters calculated from the realignment pre-processing step were included as regressors of no interest. Regressors were convolved with the default SPM5 canonical Hemodynamic Response Function (HRF) and estimated using classical ReML (Restricted Maximum Likelihood).

The resultant parameter estimates for each regressor (at each voxel) were compared using t-tests to establish the significance of differences in activation between conditions. The main effects of interest were *Hated face>Neutral faces* and *Neutral faces>Hated face*. We were also interested in *All faces>Baseline*. Contrast images for these effects for each subject were entered into a random effects (second-level) analysis, reported below; this included the PHS score for each subject as a covariate.

## Results

Unless otherwise stated, we report probabilities at significance p≤0.05 which have been corrected family-wise for multiple comparisons using Gaussian random field theory [8], either over the whole brain, or restricted to a defined search volume. Some activations were significant only at cluster level; in these cases the underlying voxel-level probability threshold which generated the clusters is always p<0.00025 (uncorrected) and this is the displayed threshold in all figures. Voxel co-ordinates are quoted in millimetres in MNI space.

### Activations with faces

**Figure 2:**
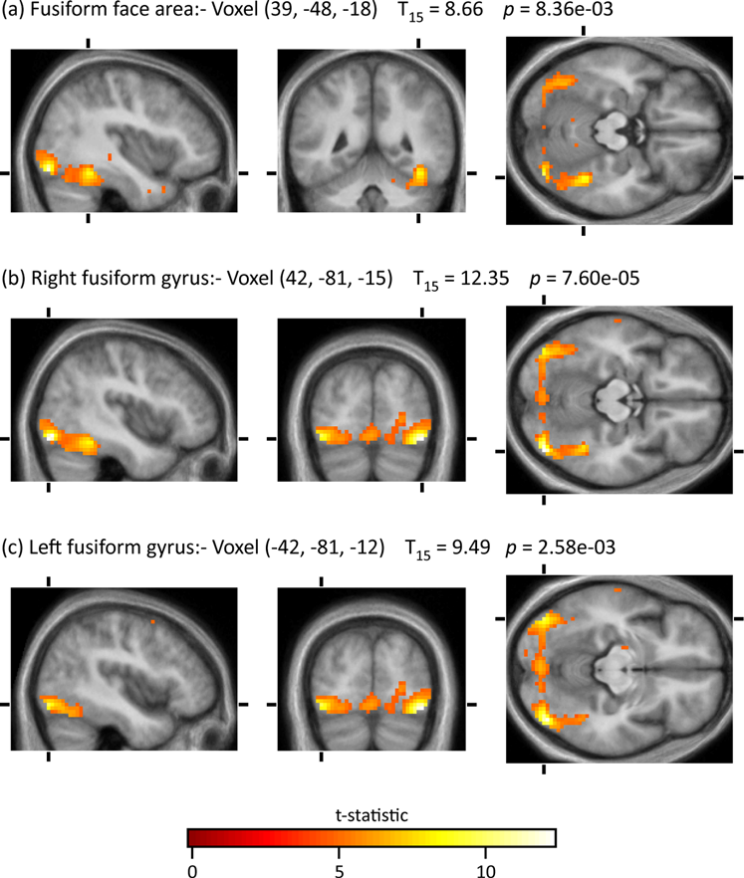
Activations for the contrast *All Faces>Baseline*. Reported probabilities at voxel level are corrected family-wise for multiple comparisons over the whole brain volume.

Given that we are dealing in this work with a sentiment that has not been studied before, we used the contrast *All faces>Baseline* to learn whether it revealed activity in the part of the fusiform gyrus that has been implicated in the perception of faces, and thus validate the activity produced by the main contrast (*Hated faces>Neutral faces*) (Figure 2). The contrast led to activity in the fusiform face area at (**a**) (39, −48, −18), almost identical to the locus that has been pinpointed in previous studies of face perception. In addition, it produced activity bilaterally elsewhere in the fusiform gyrus, at (**b**) (42, −81, −15) and (**c**) (−42, −81, −12), close to the visual motion area, V5. Activity in the latter area has been observed in other studies that have used faces in imaging experiments (e.g. Hadjikhani et al 2008 [11]).

### Activation with hated faces

**Figure 3:**
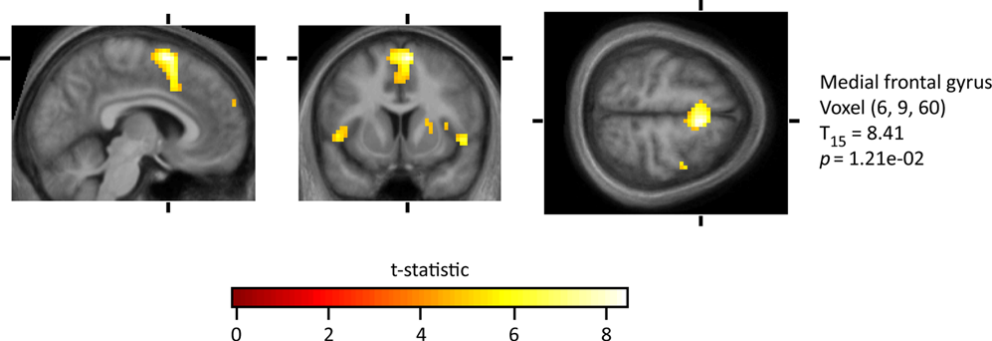
Activation for the contrast *Hated faces>Neutral faces*. The reported voxel-level probability has been corrected family-wise for multiple comparisons over the whole brain volume.

**Figure 4:**
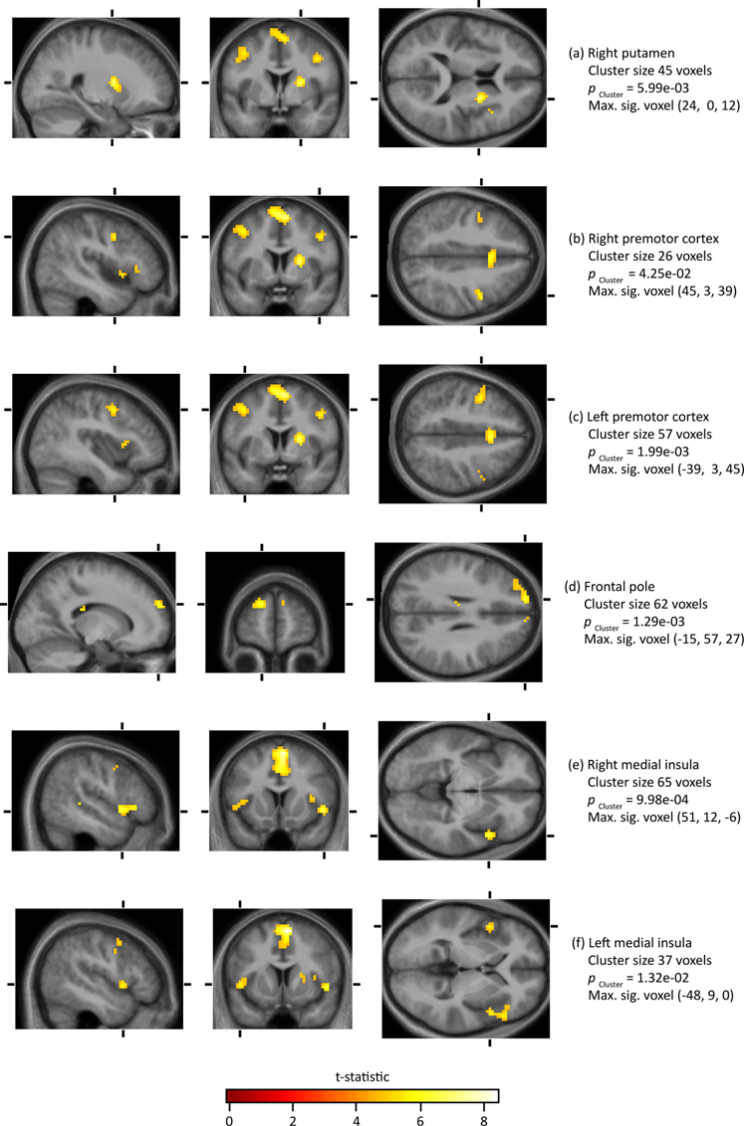
Clusters of activation for the contrast *Hated face>Neutral faces*. The statistical threshold was set at *p*≤0.05 at the cluster level, corrected for multiple comparisons, with an underlying voxel-level threshold of *p*≤0.00025, as displayed.

Our principal interest was to learn whether there are any cortical areas that are especially active in the contrast *Hated face>Neutral face*. Across all seventeen subjects, there was a voxel level activation in the medial frontal gyrus (at 6, 9, 60) (Figure 3). This was part of a cluster of 269 voxels (with an underlying voxel-level threshold of p<0.00025). In addition, there were 6 activations significant at the cluster level. The maximally significant voxel in each cluster was located as follows: (**a**) the right putamen (at 24, 0, 12); (**b,c**) bilaterally in the premotor cortex at (45, 3, 39) and (−39, 3, 45); (**d**) in the frontal pole (at −15, 57, 27) and (**e,f**) bilaterally in the medial insula (at 51, 12, −6 and −48, 9, 0) (see Figure 4)

**Figure 5:**
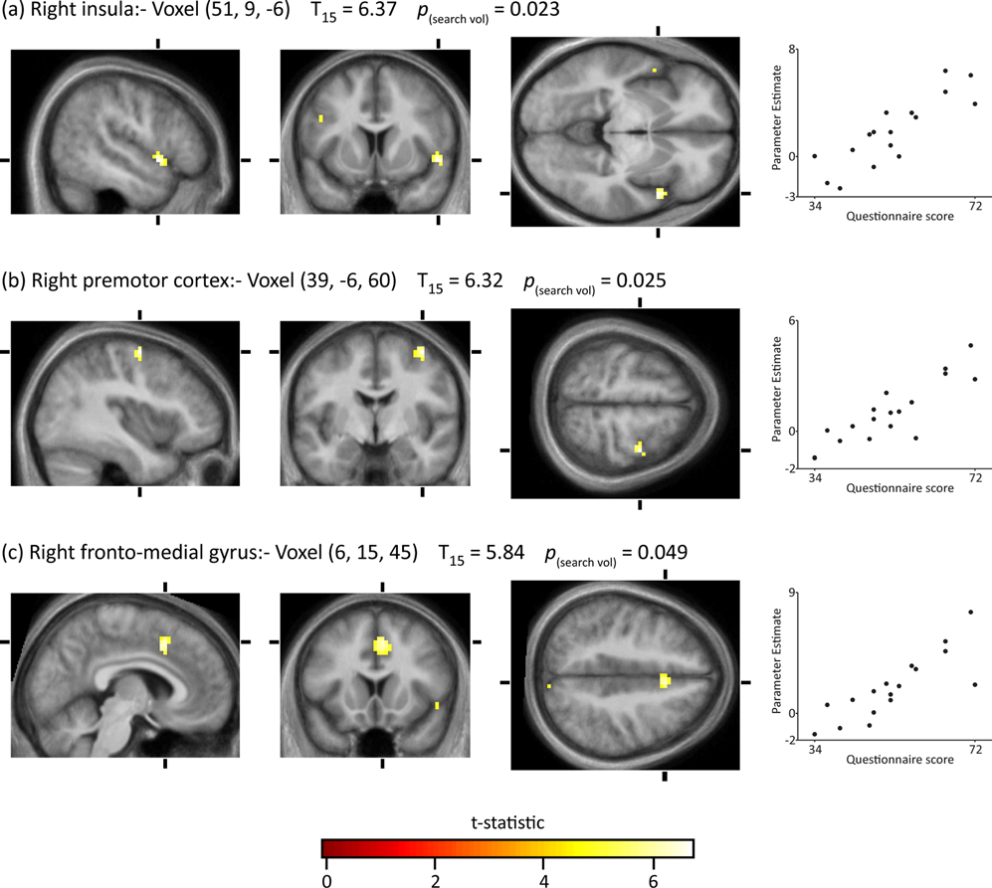
Voxels covarying with hate questionnaire score. A search volume of 5,225 voxels was defined using the t-statistic for the effect *Hated face>Neutral faces* with the statistical threshold set at *p*≤0.01 (uncorrected). Within this search volume voxels were identified where the effect *Hated face>Neutral faces* covaried with the hate questionnaire score. The voxel-level statistical threshold was set at *p*≤0.05, family-wise corrected for multiple comparisons within the search volume . The graphs in the right hand column plot the parameter estimate of *Hated face>Neutral faces* against the questionnaire score at each voxel.

We wanted, next, to learn whether there was a relationship between brain activity and the degree of hate as determined from the total scores obtained from the PHS. To do so, we entered the hate questionnaire score for each subject as a covariate in the GLM for the second level analysis. A search volume of 5,225 voxels was defined using the *t*-statistic for the effect *Hated face>Neutral faces* (a contrast orthogonal to the PHS covariate) with an uncorrected statistical threshold of *p*≤0.01. Within this search volume there were three voxels where the effect *Hated face>Neutral face* co-varied linearly and significantly (*p*_(search vol.)_≤0.05) with the hate questionnaire score. They were: (**a**) in the right insula at (51, 9, −6), (**b**) in the right premotor cortex at (39, −6, 60) and (**c**) the right fronto-medial gyrus (6, 15, 45) (Figure 5). Voxel (a) lies within the cluster in Figure 4e; voxel (c) lies within the cluster shown in Figure 3. The locus of activity in the right premotor cortex was 23 mm from the maximally active voxel in Figure 4b. In all three loci, the change in parameter estimates was directly proportional to the hate score (Figure 5, right).

No voxels were found which showed a second order polynomial (quadratic) relationship with the hate score, nor were any significantly covarying voxels found in the voxels excluded by the search volume.

### Deactivations with hated faces

**Figure 6:**
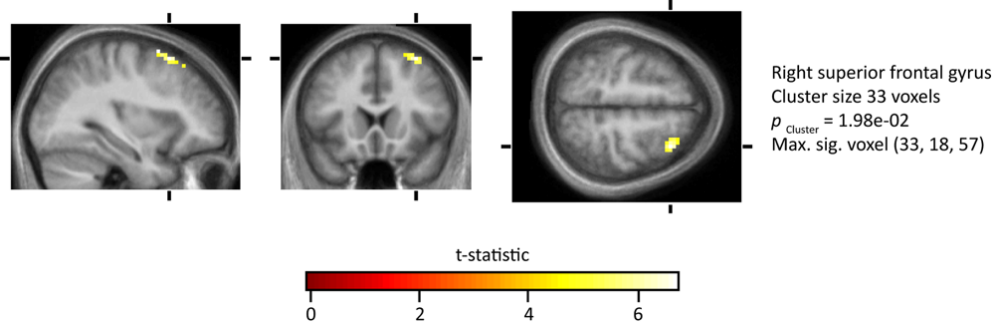
Clusters of activation for the contrast *Neutral faces>Hated faces*. The statistical threshold was set at *p*≤0.05 at the cluster level, corrected for multiple comparisons, with an underlying voxel-level threshold of *p*≤0.00025, as displayed.

In the contrast *Neutral faces>Hated Faces*, there was a cluster-level deactivation with a maximally significant voxel situated in the right superior frontal gyrus at (33, 18, 57) (Figure 6).

## Discussion

To simplify our task in approaching so complex a sentiment, we concentrated on the sentiment of hate directed against an individual. Even within such a limit, the problem has many facets that this initial study could not address. Hatred against an individual may be seemingly irrational and rooted in remote anthropological instincts. Hate based on race or religion would probably fall under this heading. On the other hand, an individual may trace the hatred to a past injustice and hence find a justifiable source for it. There are no doubt many other ways in which the sentiment can be sub-categorized. But it seemed to us that concentrating on individual hate, regardless of the categories to which it could potentially be assigned, had the merit of revealing at least a basic network in the brain and thus acting as a template for future, more specialized and sophisticated studies.

Our studies did indeed reveal a basic pattern. As far as we can determine, it is unique to the sentiment of hate even though individual sites within it have been shown to be active in other conditions that are related to hate. The network has components that have been considered to be important in (a) generating aggressive behavior and (*b*) translating this behavior into motor action through motor planning. Finally, and most intriguingly, the network involves regions of the putamen and the insula that are almost identical to the ones activated by passionate, romantic, love.

It is important to note that the pattern revealed is distinct from that of other, closely related, emotions such as fear, anger, aggression and danger, even though it shares common areas with these other sentiments. Thus, the amygdala which is strongly activated by fear (Noesselt et al. 2005 [9], Morris et al. 2002 [10], Hadjikhani et al. 2008 [11]) and by aggression (Beaver et al., 2008 [12]) was not activated in our study. Nor were the anterior cingulate, hippocampus, medial temporal regions, and orbitofrontal cortex, apparently conspicuous in anger and threat (Denson et al. 2008 [13]; Bufkin and Luttrell 2007 [14]; McClure et al. 2004 [15]), evident in our study. It would thus seem that, though these sentiments may constitute part of the behaviour that results from hatred, the neural pathways for hate are distinct.

One region of activation in our study, involving multiple foci, lies in the frontal cortex, both medially and laterally. Numerous studies have activated one part or another of this relatively large expanse of cortex. What seems not to be in doubt is that this cortical zone involves the premotor cortex, a zone that has been implicated in the preparation of motor planning and its execution (Hanakawa et al. 2008) [16]. We hypothesize that the sight of a hated person mobilizes the motor system for the possibility of attack or defense. In addition, the involvement of the frontal pole is in a location which Ramnani and Miall (2003) [17] consider to be critical in predicting the action of others, arguably an important feature when confronted by a hated person. Another forebrain site that was active in our study and which has been implicated in motor planning, though seemingly in an affective context, is the right putamen, a structure that has also been implicated in the perception of contempt and disgust (Phillips et al. 1998 [18]; Sprengelmeyer et al. 1998 [19]; Sambataro et al, 2006 [20]; Thielscher and Pessoa 2007 [21]) and fear (Surguadze 2003 [22]), possibly within an aggressive context since dopamine turnover level is apparently higher in the putamen of aggressive mice (Tizabi et al., 1980 [23]). Moreover, damage to the putamen and insula apparently compromises a patient's ability to recognize signals of disgust (Calder et al. 2000 [24]). Animal studies suggest that the putamen may constitute part of the motor system that is mobilized in the context of hate. It contains neurons that are active in phases preparatory to motor acts (Alexander and Crutcher 1990 [25]) and has been shown to be active in conditions in which cognitive planning is required to trigger a motor act (Monchi et al 2006 [26]; Boecker et al. 2008 [27]).

We note with considerable interest that the parts of the right putamen and the medial insula activated in this study correspond closely to the parts activated in our earlier study of romantic love (Bartels & Zeki 2004 [2]). The insula has been implicated in a variety of functions and of interest in this context is its involvement in expressions of disgust and the appraisal of disagreeable stimuli (Phillips et al. 1997 [28]). Reiman et al. (1997) [29] suggest that the insula may be involved in responses to distressing sensory stimuli, of which a hated face would be one example but there are also conditions in which a loved face may constitute a distressing signal. The putamen could also be involved in the planning of aggressive motor acts within the context of romantic love – for example, when a rival presents a danger. It is difficult in the present state of knowledge to be more precise about the nature of the links between the parts of the insula and putamen that are active in these two different conditions. What is not in doubt is that there is, in the behavioural sense, a strong link between the two sentiments and one can easily transmute into the other.

It is noteworthy that there was a linear relationship between the hate scores and the parameter estimates for the contrast *Hated face>Neutral faces*. Two of the three activations were located within significantly active clusters in the *Hated face>Neutral face* contrast, while the third one was located in close vicinity of the active cluster in the right premotor cortex. Such a linear relationship is of considerable interest in adding further to the accumulating evidence that subjective mental states can be quantified in terms of cortical activity (see for example Elliot et al., 2003 [30] and Knutson et al. 2001 [31]; Kawabata and Zeki 2004 [4]). The general pattern is also similar to other studies of subjective mental states, in that activity in only some of the areas is linearly related to declared subjective mental states.

### Deactivations

Equally interesting was the observed pattern of deactivation. Unlike the study of romantic love, when we observed a deactivation pattern that included frontal, temporal and parietal regions of the cerebral cortex (Bartels & Zeki 2000 [1]), the deactivation pattern observed in this study was much more restricted. It involved the right superior frontal gyrus. The deactivated locus in the frontal cortex is close in position to the one which previous studies had shown to be negatively correlated with obsessive-compulsive states (McGuire, Bench, Frith, Marks et al. 1994 [32]), a deactivation hypothesized to relate to a shift in attention from extrapersonal space to an internal experience associated with anxiety.

This difference in the extent of deactivated cortex, compared to the deactivated cortex in the context of romantic love, may seem surprising, since hate too can be an all consuming passion. But whereas in romantic love, the lover is more likely to be less critical and judgmental regarding the loved person, it is more likely that in the context of hate the hater may want to exercise judgment in calculating moves to harm, injure or otherwise extract revenge.

In summary, our results show that there is a unique pattern of activity in the brain in the context of hate. This pattern, while being distinct from that obtained in the context of romantic love, nevertheless shares two areas with the latter, namely the putamen and the insula. This linkage may account for why love and hate are so closely linked to each other in life.

## Supporting Information

File S1 Hate Questionnaire
(0.04 MB DOC)
