# Supplementary material for: Neural Correlates of Hate
Source: PLoS One. 2008 Oct 29;3(10):e3556. doi: 10.1371/journal.pone.0003556 (PMC2569212; doi:10.1371/journal.pone.0003556)
Supplement: File S1 — Hate Questionnaire (0.04 MB DOC) [file pone.0003556.s001.doc]

Supporting Information

*Hate questionnaire*

A questionnaire was devised to assess each subject’s feelings about the hated person. The questionnaire was based on Sternberg’s [4] triangular theory of the structure of hate. Sternberg proposes the following three elements of hate:-

A/ Negation of Intimacy (Distancing) in Hate: Repulsion and Disgust

B/ Passion in Hate: Anger-Fear

C/ Decision-Commitment in Hate: Devaluation-Diminution Through Contempt

For each of these components A, B and C we construct three negative statements (1/2/3) and one positive statement (4) as below:-

A1/ I don’t want X anywhere near me.

A2/ The world would be a better place if X had never existed.

A3/ Any time spent with X is a waste of time.

A4/ I would like to interact with X.

B1/ I cannot control my hatred for X.

B2/ I would like to do something to hurt X.

B3/ I have violent thoughts about X.

B4/ I have kind thoughts for X.

C1/ X is scum.

C2/ X is a low class type of person.

C3/ X does not deserve any consideration or compassion.

C4/ X is a very nice person.

There are twelve statements in all.  In each statement “X” represents the hated person. For each statement the subject must pick one of the following seven evaluations;

“Strongly agree” “Agree” “Mildly agree” “Don't know” “Mildly disagree” “Disagree” “Strongly disagree”

The negative statements (1/2/3 for each component) are scored from zero for "Strongly disagree" to six for "Strongly agree".  The positive statements (4 for each component) are scored in the opposite sense.  Therefore the questionnaire can result in a total "hate" score ranging from 0 (minimum hate) to 72 (maximum hate).

The questionnaire was completed by each subject once during the laboratory visit and once during the scanning visit. On each occasion the questions were presented in a different randomised order.

The hate questionnaire scores had a possible range of 0 to 72. Subjects scores during the laboratory visit ranged from 36 to 66. Scores obtained directly after scanning (which were used in the second level analysis) ranged from 34 to 72. There were substantial differences (both positive and negative) between some of the scores obtained during the lab visit and those obtained directly after the scan, the largest difference being a drop of 16 in the second visit score.

*Processing of face images*

Four images of faces were selected from the images provided by each subject, one of the hated person, three others of people for whom the subject had neutral feelings. The images were either full face or three-quarter profile. The faces were rotated if necessary to align them with the vertical axis. As far as possible the expressions (e.g. smiling, serious etc...) were matched for each subject. The images were digitized and an image-editing program (Adobe® Photoshop® CS2) was used to remove any obscuring articles such as earrings, scarves etc... A square containing just the head was cropped from each image. Each of these square images was then resized to the pixel dimensions of the smallest image, to roughly normalise the spatial frequency of the four images. The background detail was then replaced with a flat mid-grey tone.

When the images were finally displayed to the subject during the second session some further normalisation was imposed:-

i/ The images were converted to greyscale.

ii/ The images were individually scaled so that each face had the same surface area.

iii/ The mean brightness of each face was the same.

iv/ The four faces were roughly matched in contrast. The frequency distribution of individual pixel intensities was calculated for each image. The overall intensities of the image were stretched linearly about the mean so that the intensity difference between the 5^th^ and 95^th^ percentiles of the frequency distribution was the same for each image. The contrast was maximised for each set of four images so that somewhere in one of the four images there was either a pixel of the darkest (0.23 cd/m^2^) or of the lightest (34.1 cd/m^2^) possible intensity afforded by the projection system.

In order to perform the intensity and contrast normalisations the gamma profile of the projector used to display the images during the second session was measured using a PHOTORESEARCH® PR®-650 spectra-colorimeter. The normalisation was implemented using custom-written scripts for the MATLAB® (©The MathWorks Inc. ) programming environment.

*Stimulus design*

Stimuli were generated using Cogent 2000 (http://www.vislab.ucl.ac.uk/Cogent2000) and Cogent Graphics (http://www.vislab.ucl.ac.uk/CogentGraphics).

Each subject undertook either two or three identical stimulus sessions. During each session a flat grey background (intensity 9.06 cd/m^2^) is presented. Such a plain grey background will be denoted here as “blank”.

Scanner acquisition commences and the session begins with an initial blank period of approximately 20 seconds during which the first six brain volumes are discarded to allow T1 equilibration effects to subside.

Then the faces are presented. As described earlier the faces are normalised for surface area, average brightness (approx 10 cd/m^2^) and contrast. The contrast measure was the intensity difference between the 5^th^ and 95^th^ percentiles of the frequency distribution of intensities of pixels in the image. This remained constant within the four images shown to each subject, but varied across subjects from 13.3 cd/m^2^ to 22.1 cd/m^2^. This variation occurred because the contrast was maximised for each set of four face images. For each subject the visual area of the four faces was constant, but there were differences between subjects due to differences in the images and due to the precise geometry of scanner, projector and subject. Across all subjects the smallest visual area was 0.0132 steradians and the largest was 0.0269 steradians. Across all subjects face height varied from 8.1° to 13.1° (0.14 to 0.23 radians) and face width varied from 6.0° to 10.5° (0.10 to 0.18 radians).

A face is presented for 16.07 seconds and this is followed by a blank interval of 2.07 seconds. Sometimes a blank period of 16.07 seconds is displayed instead of a face. Blank periods are included to increase the proportion of baseline acquisition during the scanning session.

The session finishes with a terminal blank period of 30 seconds during which time the scanner continues to acquire decaying BOLD signal after the end of the stimulus period.

Faces were presented according to the following sequence of presentations:-

Intro:1-2-3-H-B-2-H-1-3-B-3-1-H-2-B-H-3-2-1-B-2-1-H-3-B-1-3-2-H-B-H-2-3-1-B-3-H-1-2-B:Outro

| Intro: | = Initial blank period of ca 20 seconds |
| --- | --- |
| 1 | = Neutral face 1 presented for 16.07 seconds |
| 2 | = Neutral face 2 presented for 16.07 seconds |
| 3 | = Neutral face 3 presented for 16.07 seconds |
| H | = Hated face presented for 16.07 seconds |
| B | = Blank presented for 16.07 seconds |
| - | = Blank interval of 2.07 seconds |
| :Outro | = Terminal blank period of 30 seconds |

Total stimulus duration including Intro: and :Outro was 12’ 53”. The sequence is symmetrically balanced. It can be broken down into eight blocks of five presentations.:-

1-2-3-H-B- 2-H-1-3-B- 3-1-H-2-B- H-3-2-1-B-

2-1-H-3-B- 1-3-2-H-B- H-2-3-1-B- 3-H-1-2-B

Each block contains the elements 1, 2, 3, H and ends with B. In the complete sequence each element (1, 2, 3 and H) starts a block twice, occupies position 2 twice, position 3 twice and position 4 twice. Furthermore each element is both preceded and followed by each of the other elements exactly twice during the complete sequence.
